# Targeting ornithine decarboxylase (ODC) inhibits esophageal squamous cell carcinoma progression

**DOI:** 10.1038/s41698-017-0014-1

**Published:** 2017-04-27

**Authors:** Wei He, Eunmiri Roh, Ke Yao, Kangdong Liu, Xing Meng, Fangfang Liu, Penglei Wang, Ann M. Bode, Zigang Dong

**Affiliations:** 1 The Hormel Institute, University of Minnesota, Austin, MN 55912 USA; 2 The First Affiliated Hospital of Zhengzhou University, Zhengzhou, 450052 China; 3 Basic Medical College, Zhengzhou University, Zhengzhou, 450001 China; 4 The China-US (Henan) Hormel Cancer Institute, Zhengzhou, 450008 China

## Abstract

To explore the function of ornithine decarboxylase in esophageal squamous cell carcinoma progression and test the effectiveness of anti-ornithine decarboxylase therapy for esophageal squamous cell carcinoma. In this study, we examined the expression pattern of ornithine decarboxylase in esophageal squamous cell carcinoma cell lines and tissues using immunohistochemistry and Western blot analysis. Then we investigated the function of ornithine decarboxylase in ESCC cells by using *shRNA* and an irreversible inhibitor of ornithine decarboxylase, difluoromethylornithine. To gather more supporting pre-clinical data, a human esophageal squamous cell carcinoma patient-derived xenograft mouse model (C.B-17 severe combined immunodeficient mice) was used to determine the antitumor effects of difluoromethylornithine in vivo. Our data showed that the expression of the ornithine decarboxylase protein is increased in esophageal squamous cell carcinoma tissues compared with esophagitis or normal adjacent tissues. Polyamine depletion by *ODC shRNA* not only arrests esophageal squamous cell carcinoma cells in the G2/M phase, but also induces apoptosis, which further suppresses esophageal squamous cell carcinoma cell tumorigenesis. Difluoromethylornithine treatment decreases proliferation and also induces apoptosis of esophageal squamous cell carcinoma cells and implanted tumors, resulting in significant reduction in the size and weight of tumors. The results of this study indicate that ornithine decarboxylase is a promising target for esophageal squamous cell carcinoma therapy and difluoromethylornithine warrants further study in clinical trials to test its effectiveness against esophageal squamous cell carcinoma.

## Esophageal cancer: African sleeping sickness drug shows activity against tumors

Blocking an enzyme involved in the cellular synthesis of essential compounds called polyamines could help treat esophageal cancer. Zigang Dong from the University of Minnesota’s Hormel Institute, USA, and colleagues showed that this enzyme, called ornithine decarboxylase (ODC), is expressed at elevated levels in tumor tissues taken from patients with esophageal squamous cell carcinoma. The researchers blocked ODC activity in esophageal cancer cells using either RNA interference techniques or a drug called difluoromethylornithine (DFMO). In both cases, the treatment suppressed further growth and induced cell death. DFMO treatment also reduced the size and weight of tumors in mice implanted with human patient-derived esophageal cancer tissue. The findings point DFMO, which is already used as a medication to treat African sleeping sickness and excessive hair growth, as a potential therapy for cancer patients.

## Introduction

Esophageal cancer is the 8th most common cancer worldwide with an estimated 456,000 new cases each year.^1^ Esophageal squamous cell carcinoma (ESCC) is the dominant histological type and accounts for 80% of esophageal cancers.^2^ With the increased understanding of cancer biology, more and more targeted drugs, such as gefitinib,^3^ cetuximab^4^ or imatinib administration^5^ have been approved for clinical treatment because of their higher efficacy and lower toxicity compared with traditional chemotherapy. However, an effective therapeutic drug targeting ESCC has not yet been developed. ESCC is the 4th leading cause of cancer death in China and 7th in the world because of its ability to develop chemoresistance and tendency to metastasize.^6^ Even for patients with early stage ESCC, adjuvant therapy cannot prolong their overall survival significantly compared with surgery alone.^7^

Polyamines are a group of small aliphatic polycations derived from amino acids and are present in all living organisms. The ubiquity of polyamines indicates their indispensable role in key cellular processes, such as cell growth,^8^ proliferation,^9^ apoptosis^10^, and gene expression.^11^ Aberrant accumulation of polyamines is associated with various pathological consequences, including cancer.^11^ Ornithine decarboxylase (ODC) is the first rate-limiting enzyme in the polyamine biosynthesis pathway in mammals and its intracellular concentration is tightly controlled. ODC activity is induced in response to cell growth stimuli, and is highly expressed in diseases such as inflammation and cancer. ODC is considered to be a potential oncogene because its over-expression can transform mammalian cell lines,^12^ indicating that ODC is not only a biomarker for cancer but also a potential target for cancer therapy. Anti-cancer research, including bench work and clinical research, targeting ODC has yielded promising results.^13, 14^ However, the role of ODC in ESCC development is still unclear. The Wnt signaling pathway is activated in most ESCC cases^15^ and the *Myc* loci comprise the most significant regions of amplification.^16^ *Myc* has been implicated as a reasonable indicator of the accumulation of various activated and inactivated genes involved in the development of ESCC,^17^ suggesting *Myc* expression acts as a driver event of ESCC. As a physiological transcriptional target of c-*Myc*,^18^ ODC reportedly plays an important role in ESCC development and progression.

In the present study, we examined the expression pattern of ODC in ESCC cell lines and tissues. Then we investigated the functions of ODC in ESCC development by using *shRNA* and an irreversible inhibitor of ODC, difluoromethylornithine (DFMO). Our data showed that ODC expression was up-regulated in human ESCC tissues and ESCC progression could be attenuated by suppressing ODC activity, indicating that ODC might be a promising target for ESCC therapy.

## Results

### The expression of ODC is up-regulated in ESCC tissues

**Fig. 1:**
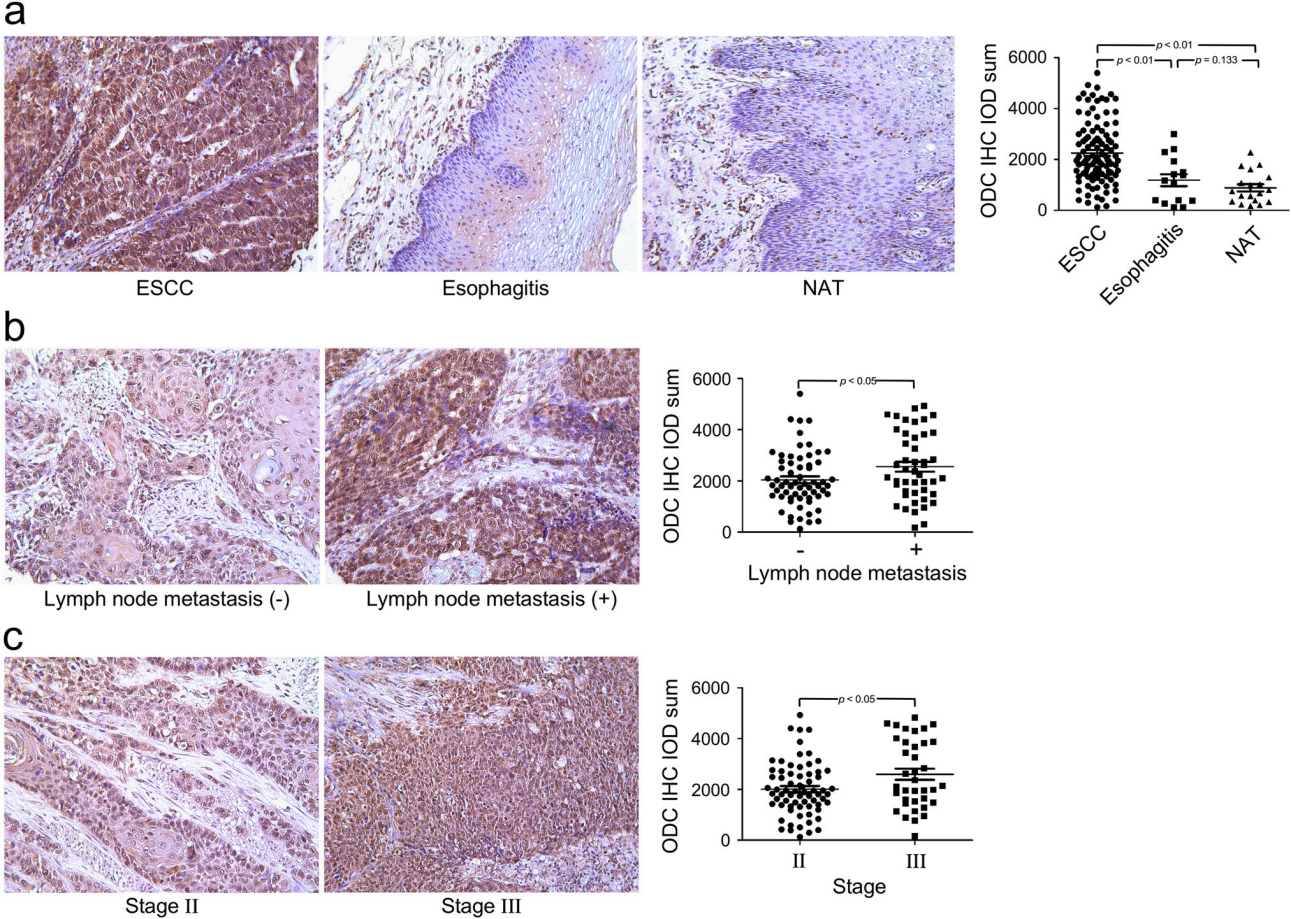
ODC expression is up-regulated in ESCC. **a** The ODC immunohistochemical IOD value is significantly higher in ESCC compared to esophagitis or NAT. The ODC protein expression level in ESCC is significantly correlated with **b** status of lymph node metastasis and **c** clinical stage. Significant differences were determined using the Student’s *t* test

Up-regulated ODC expression has been reported in various solid cancers, including skin cancer,^19^ gastric cancer,^20^ neuroblastoma,^21^ and colon cancer.^22^ In the present study, we explored the expression pattern of ODC in ESCC tissues by immunohistochemistry (IHC). ODC immunostaining was observed in both nucleus and cytoplasm (Fig. 1). Among 110 evaluable ESCC cases, positive staining for ODC was observed in 93.6% of the tissues (103/110). In esophagitis and normal adjacent tissues (NAT), ODC positive staining was 60.0% (9/15) and 52.6% (10/19), respectively (Supplementary Fig. 1). Quantitative analysis showed that the ODC immunostaining integrative optical density (IOD) value was 2255 ± 115 for ESCC, which was significantly higher than the value for esophagitis (1187 ± 236, *p* < 0.01) or NAT (888 ± 140, *p* < 0.01; Fig. 1a). The correlations between clinicopathological features and ODC expression in the primary ESCC were determined (Supplementary Table 2). The ODC expression level in ESCC was significantly correlated with lymph node metastasis status (*p* = 0.02, Fig. 1b) and clinical stage (*p* = 0.02, Fig. 1c), but was not associated with age, gender, or tumor histological grade.

### Knocking down ODC expression attenuates proliferation and anchorage-independent growth of ESCC cells

**Fig. 2:**
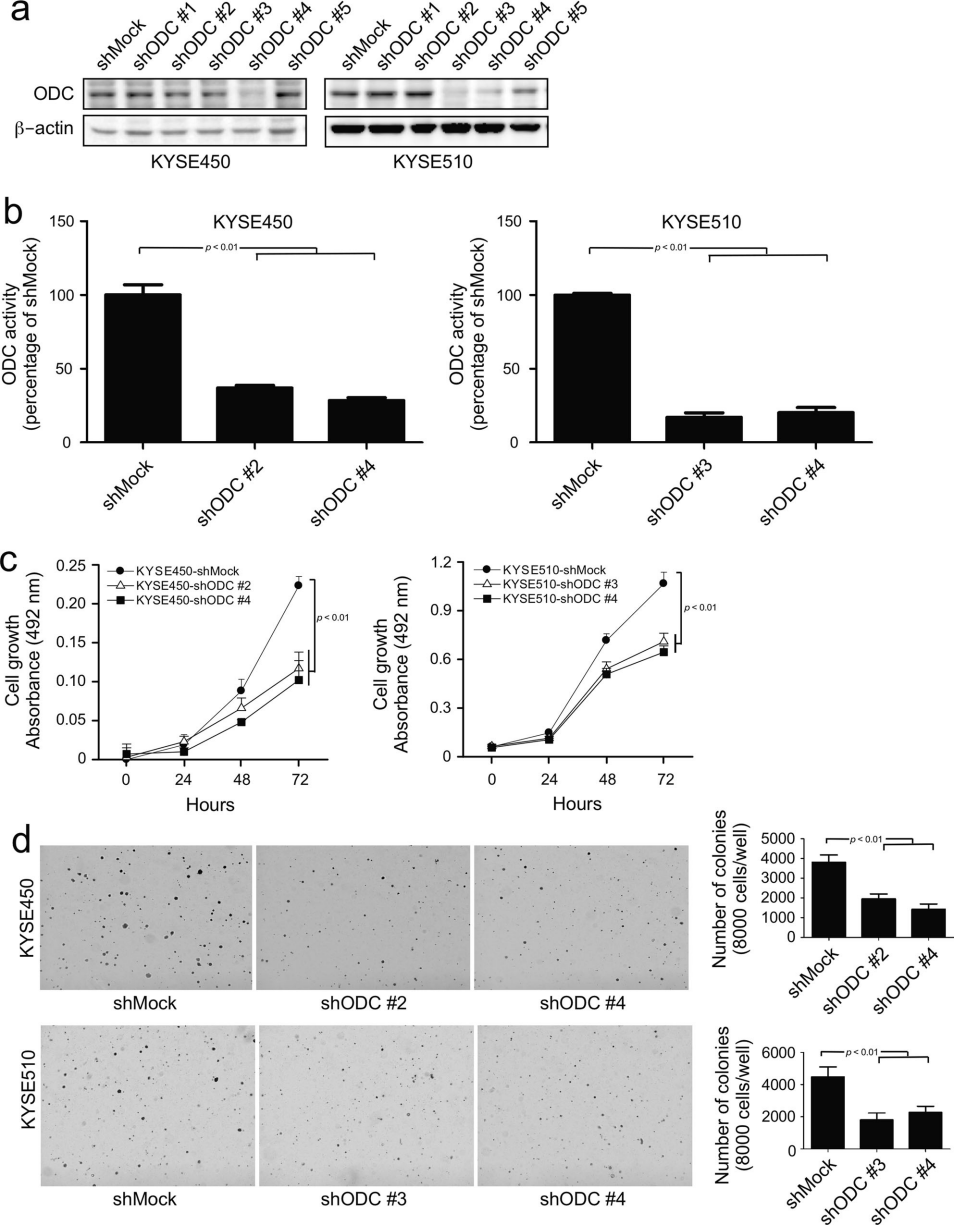
Silencing ODC expression by *shRNA* suppresses anchorage-dependent and anchorage-independent ESCC cell growth. **a** ODC expression was analyzed by Western blot in KYSE450 and KYSE510 cells expressing *shMock* or *shODC*. **b** ODC activity was assessed as the release of L-[1-C^14^] ornithine and results are shown as percentage of the control group (set at 100%). **c** Anchorage-dependent cell growth was measured by MTS assay. **d** For anchorage-independent growth, cells were cultured in soft agar for 3 weeks and then colonies were counted using a microscope and the Image-Pro Plus software (v.6.0) program. All data are shown as means ± S.D. of triplicate values from 3 independent experiments

To further investigate the role of ODC in ESCC progression, we introduced *shODC* into two human ESCC cell lines, KYSE450 and KYSE510, by lentiviral vector and examined the effect of ODC on proliferation and anchorage-independent growth. To select the most effective *shRNA*, we screened 5 different *shRNA* sequences targeting ODC. Our data showed that, *shODC #2* and *#4* worked well in KYSE450 cells, whereas *shODC#3* and *#4* worked well in KYSE510 cells (Fig. 2a). These *shODC* sequences were used for experiments. Knocking down ODC expression reduced ODC enzyme activity in both ESCC cell lines by more than 70% (Fig. 2b). Correspondingly, cell proliferation and colony formation in soft agar were also suppressed (Fig. 2c, d), indicating that ODC promotes the progression of ESCC and deserves further investigation.

### Knocking down ODC expression induces apoptosis and G2/M phase arrest

**Fig. 3:**
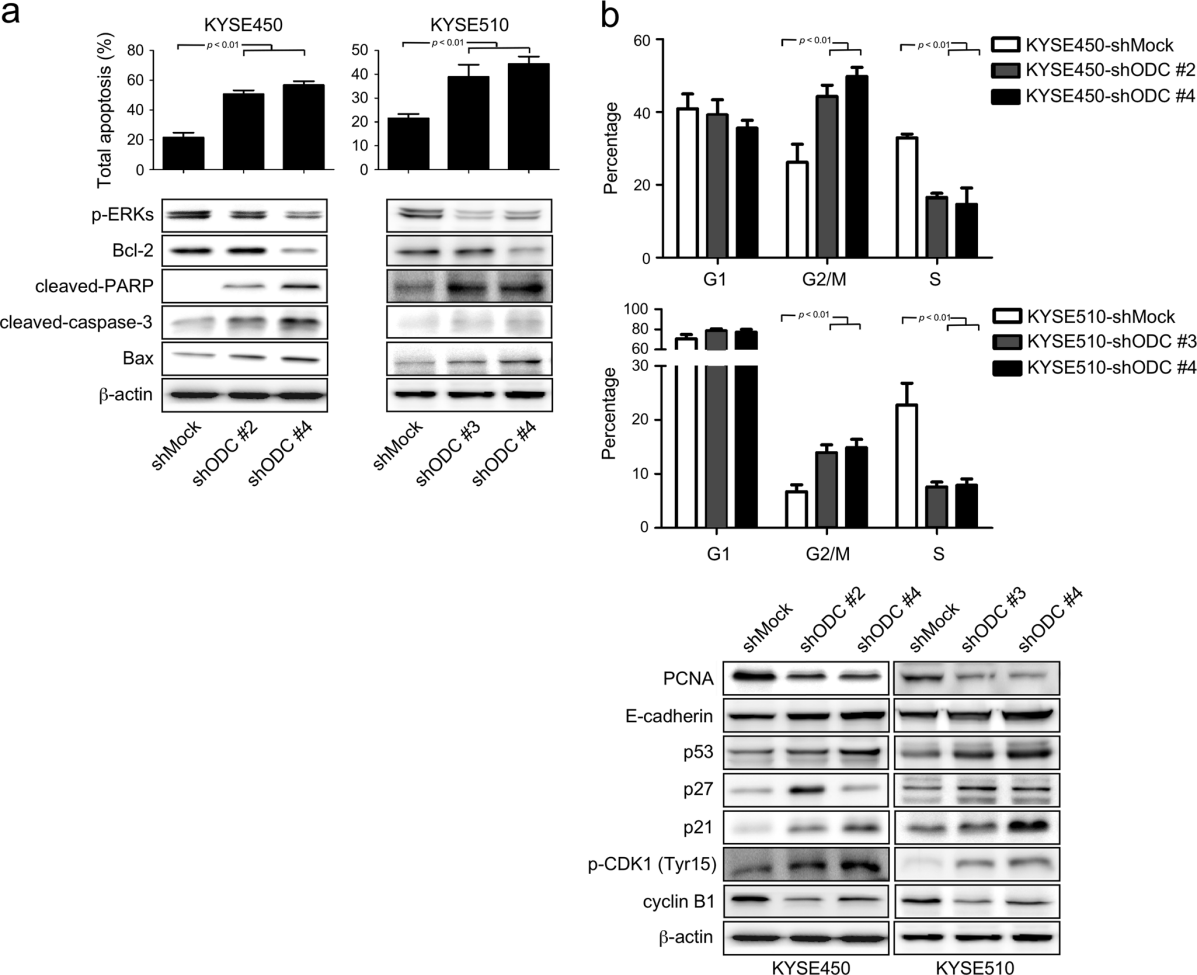
Silencing of ODC expression by *shRNA* induces apoptosis and G2/M arrest in ESCC cells. The effects of ODC on **a** apoptosis and **b** cell cycle were analyzed by flow cytometry (*upper panels)*. The expression of markers associated with cell cycle and apoptosis were analyzed by Western blot (*lower panels)*. All data are shown as means ± S.D. of triplicate values from 3 independent experiments

To explain the basis of ODC as a tumor promoter in ESCC progression, we examined apoptosis and cell cycle in *shODC*-infected ESCC cells. Our results showed that knocking down ODC expression with *shODC* significantly increased total apoptosis of both KYSE450 and KYSE510 cell lines (Fig. 3a). In addition, compared to *shMock, shODC* expression also induced G2/M phase arrest and decreased the percentage of cells in S phase in both cell lines (Fig. 3b). We also compared apoptosis- (Fig. 3a) and cell cycle-related (Fig. 3b) markers by Western blot in *shMock* and *shODC-*expressing KYSE450 and KYSE510 cells. Our results showed that *shODC* was generally associated with increases in the expression levels of cleaved caspase 3, cleaved PARP, Bax, p53, p27, p21, E-cadherin, and phosphorylated CDK1 (Tyr15). In contrast, the expression levels of phosphorylated ERK1/2, Bcl-2, PCNA and cyclin B1 were mostly decreased.

### Inhibition of ODC reduces ESCC tumorigenesis in a xenograft mouse model

**Fig. 4:**
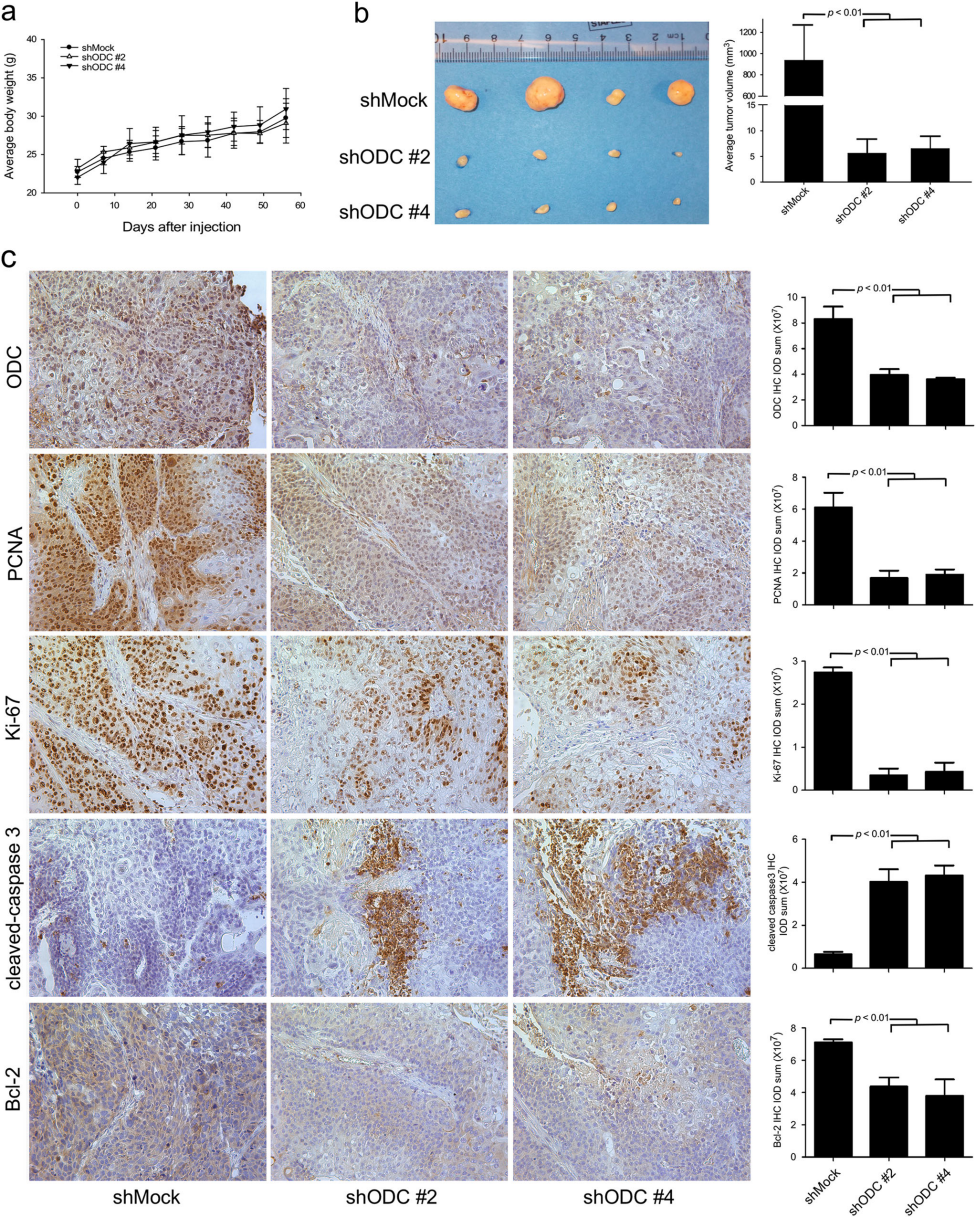
shODC suppresses the tumor-forming ability of ESCC cells. **a** After inoculation, the body weights of all mice remained stably increased. **b** *shODC* significantly suppresses KYSE450 xenograft tumor volume compared with the *shMock* group. **c** IHC analysis was performed to determine the expression levels of ODC, PCNA, Ki-67, cleaved caspase 3, and Bcl-2 in ESCC xenograft tumors expressing *shMock* or *shODC*. Representative photographs for each antibody and each group are shown. The integrated optical density (IOD) was evaluated using the Image-Pro Plus software (v.6.0) program. All data are shown as mean values ± S.D

Based on the in vitro assay results, we investigated the function of ODC in ESCC tumorigenesis in an athymic nude xenograft mouse model. Mice were divided into three groups and inoculated in the right flank with *shMock-*infected, *shODC#2-*infected, or *shODC#4*-infected KYSE450 cells (3 × 10^6^ cells per mouse, 4 mice per group). The body weight of mice increased normally with growth for the duration of the study, indicating no adverse effects from the respective inoculation (Fig. 4a). At 56 days after inoculation, when the tumor volume of the *shMock* group reached 1000 mm^3^, all experimental mice were euthanized and tumors extracted for IHC analysis (Supplementary Fig. 2). Our data showed that *shMock-*infected KYSE450 cells formed markedly larger xenograft tumors in nude mice compared to the *shODC*-infected groups (*p* < 0.01, Fig. 4b). IHC results showed that expression of ODC, Ki-67 and PCNA proliferation markers was attenuated. In addition, expression of the anti-apoptosis marker Bcl-2 was also decreased, whereas expression of cleaved caspase 3 was increased (Fig. 4c). These results confirmed that inhibiting ODC expression in ESCC cells suppresses proliferation and induces apoptosis, leading to attenuation of ESCC tumorigenesis.

### DFMO suppresses proliferation and anchorage-independent growth of ESCC cells by targeting ODC

**Fig. 5:**
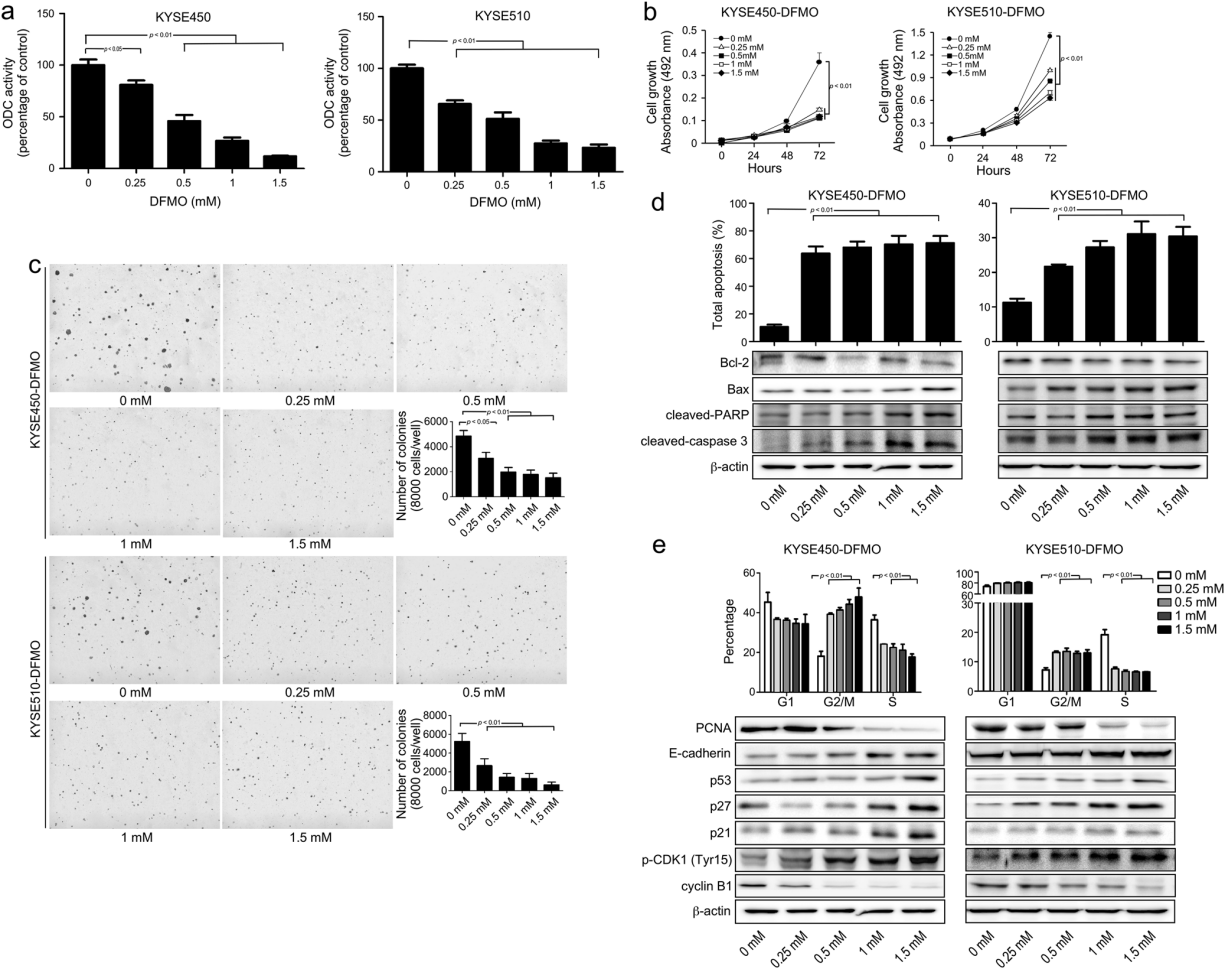
DFMO inhibits ESCC cells in vitro. **a** The effect of DFMO on ODC activity in KYSE450 and KYSE510 cells was measured as the release of CO_2_ from L-[1-C^14^] ornithine and results are shown as percentage of control group (set at 100%). **b** Anchorage-dependent cell growth was measured by MTS assay. **c** For measuring anchorage-independent growth, cells were cultured for 3 weeks with different concentrations of DFMO in soft agar and then colonies were counted using a microscope and the Image-Pro Plus software (v.6.0) program. After treatment with DFMO for 72 h, **d** total apoptosis and **e** cell cycle were analyzed by flow cytometry. The expression of markers associated with cell cycle and apoptosis was analyzed by Western blot. All data are shown as means ± S.D. of triplicate values from 3 independent experiments

Based on the important role of ODC in ESCC progression, ESCC cells were treated with DFMO, a potent substrate analog and specific irreversible inhibitor of ODC.^23^ The data showed that treatment with DFMO dose-dependently decreased ODC activity in ESCC cells (Fig. 5a). Inhibition of ODC activity was associated with suppression of proliferation and colony formation of ESCC cells (Fig. 5b, c), indicating that DFMO attenuates ESCC progression by targeting ODC.

### DFMO induces apoptosis and G2/M arrest of ESCC cells

Treatment of ESCC cells with DFMO for 72 h resulted in a dose-dependent increase in total apoptosis (Fig. 5d), G2/M arrest and a decreased S phase cell population (Fig. 5e). Western blot analysis showed that DFMO induced an increased expression of Bax, p53, p21, p27, phosphorylation of CDK1 (Tyr15), cleavage of caspase 3 and PARP and suppressed expression of PCNA, Bcl-2 and cyclin B1.

### DFMO inhibits ESCC tumorigenesis in a patient-derived xenograft (PDX) mouse model

**Fig. 6:**
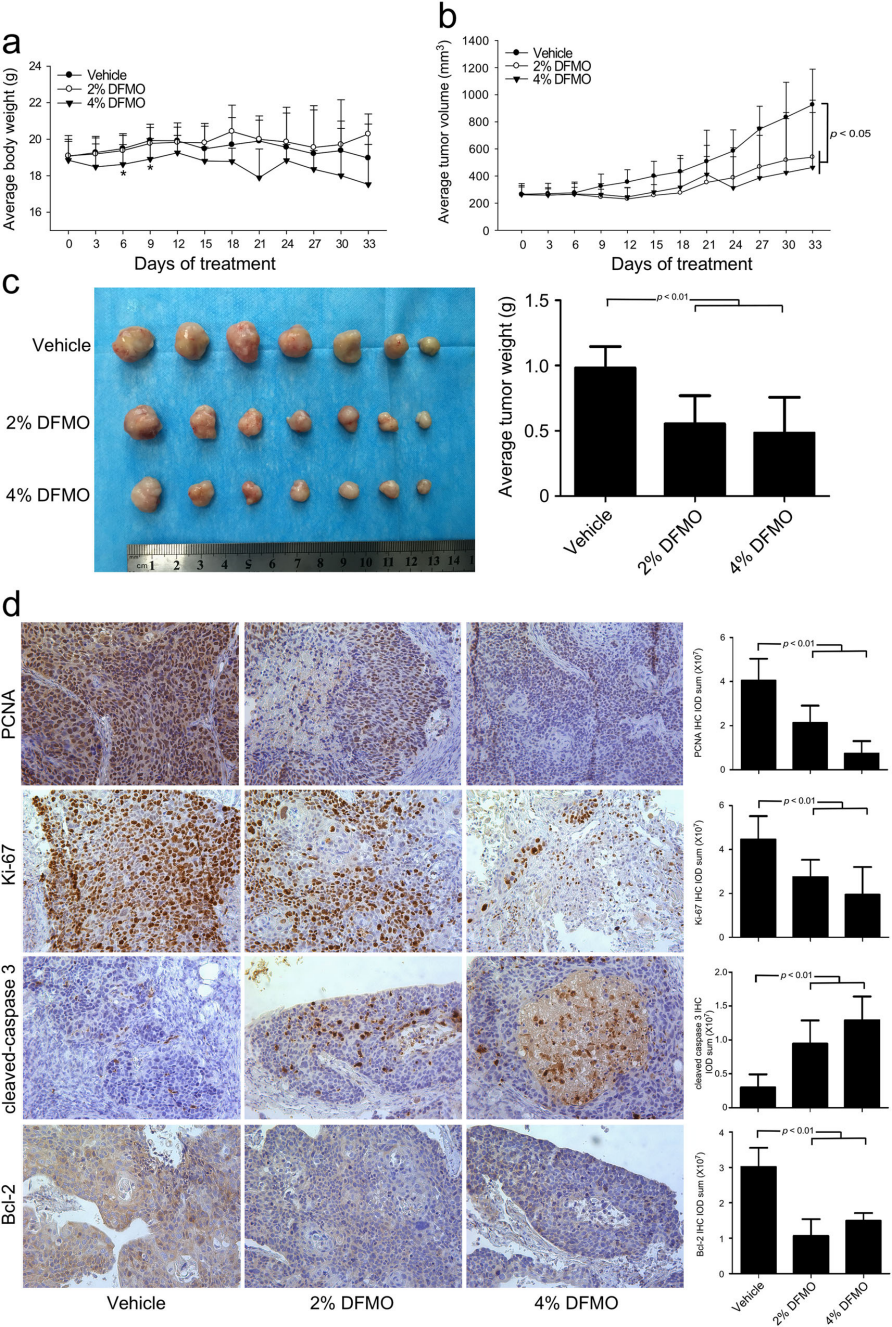
DFMO inhibits ESCC progression in a PDX model. A PDX model of mice implanted with human ESCC (EG20) were divided into three groups and treated with vehicle or 2% (v/v) or 4% (v/v) DFMO in drinking water for a total of 33 days. **a** From the 1st day of treatment, body weight and tumor volume were measured every 3 days. DFMO did not significantly reduce mouse body weight compared with the vehicle group. **b** From the 9th day of treatment, the average tumor volume of both DFMO-treated groups appeared to be significantly less than the vehicle-treated group. **c** Compared with vehicle, DFMO treatment significantly decreased the weight of the PDX tumors. **d** IHC analysis was performed to determine the expression levels of PCNA, Ki-67, cleaved caspase 3, and Bcl-2 in ESCC PDX tumors treated with vehicle or DFMO. Representative photographs for each antibody and each group are shown. The integrated optical density (IOD) was evaluated using the Image-Pro Plus software (v.6.0) program. All data are shown as mean values ± S.D

We established 3 ESCC PDX mouse models, referred to as EG20, EG5, and EG37 (Supplementary Table 1) to test the effectiveness of DFMO. None of the patients donating tumors received neo-adjuvant therapy. EG20 tumors were implanted and when the tumors reached an average volume of 250 mm^3^, mice were divided into 3 groups and treated with vehicle, 2 or 4% (v/v) DFMO in drinking water, respectively. From the 1st day of treatment, body weight and tumor volume were measured every 3 days. Results indicated that over 33 days of treatment, DFMO had no effect on mouse body weight compared with the vehicle-treated group. This indicated that even the highest dose of DFMO (4%) was tolerable and safe (Fig. 6a). Beginning at day 9, the average tumor volume of each DFMO treatment group appeared to be significantly smaller than the average volume of the vehicle-treated group (Fig. 6b). After 33 days of treatment, the tumor volume of the vehicle group reached 1000 mm^3^ and all experimental mice were euthanized and tumors extracted and weighed. Final results showed that DFMO treatment significantly decreased PDX tumor weight compared to vehicle-treated control mice (*p* < 0.01, Fig. 6c). No significant difference was observed in either tumor volume or in tumor weight between the two DFMO-treated groups (Fig. 6b, c), indicating that even the lowest dose of DFMO was effective. Similar results were obtained from the other two ESCC PDX models, EG5 and EG37 (Supplementary Fig. 3). Even the low dose of DFMO treatment (2%) could significantly suppress the progression of ESCC PDX tumors. We also examined the expression of PCNA and Ki-67 proliferation markers and cleaved caspase 3 and Bcl-2 apoptosis markers by IHC. Our results showed that DFMO treatment suppressed proliferation and induced apoptosis of ESCC cells (Fig. 6d).

## Discussion

ODC is associated with cell growth, proliferation, and transformation, and is overexpressed in various solid cancers. Moreover, a correlation between polyamine level and tumor stage is well established in colorectal cancers.^22^ One group previously showed the tumor:normal ratio of ODC mRNA expression in stage III and IV ESCC specimens is significantly higher than in stage I and II ESCC specimens (*p* = 0.043).^24^ In the present study, we disclosed the expression pattern of ODC in human ESCC. ODC immunostaining was observed in both the nucleus and cytoplasm (Fig. 1), which agrees with a previous report.^25^ The expression of ODC was at least twice as high in ESCC compared to esophagitis or NAT (Fig. 1a). Clinically, the expression level of ODC in ESCC was significantly correlated with lymph node metastasis status and TNM stage but not with either age or gender, indicating that ODC might also function as an oncogene in ESCC.

ODC affects numerous processes in carcinogenesis through its regulation of the intracellular polyamine pool. Studies focusing on transgenic mouse models have shown an essential role of polyamines in the early promotion of tumorigenesis. One group^26^ reported that elevated ODC activity is sufficient to promote skin tumor formation in the carcinogen-exposed skin of K6/ODC transgenic mice, without the addition of tumor-promoting agents. Conversely, suppression of ODC expression is associated with decreased cell proliferation, increased apoptosis and decreased expression of genes affecting tumor invasion and metastasis.^27, 28^ Our data showed that when ODC expression in ESCC cells was knocked down, both proliferation and anchorage-independent growth of ESCC cells were significantly suppressed (Fig. 2c, d).

The relationship between polyamines and apoptosis is still not well defined. Various cell lines respond differently to polyamine depletion. Yuan et al.^29^ reported that polyamine depletion prevented apoptosis of rat intestinal epithelial cells by decreasing caspase 3 and 9 activities, as well as the translocation of Bax to mitochondria, thus diminishing cytochrome c release. Similarly, Landau et al.^30^ showed that polyamine depletion in NIH3T3 cells led to cell cycle arrest but not apoptosis. In contrast, most reports demonstrated that polyamine depletion induced apoptosis in tumor cells.^31, 32^ The mitochondrial pathway is a primary pathway of apoptosis involving Bcl-2 family members, cytochrome c release and activation of caspase 3.^33^ Wildtype *p53* is a tumor suppressor gene that plays a key role in DNA damage repair. But when DNA damage is beyond repair, p53 induces Bax expression, which guides cells into apoptosis.^34^ The homodimerization of Bcl-2 leads to anti-apoptosis signaling and Bax can heterodimerize with Bcl-2 to induce apoptosis.^35^ Moreover, Bax has been reported to induce cytochrome c release that in turn activates caspase 3.^36^ Poly ADP ribose polymerase (PARP) is an important enzyme that plays a key role in DNA repair and apoptosis.^37^ Once PARP has been cleaved by caspases, apoptosis is induced.^38^ In the present study, polyamine depletion by *shODC* enhanced the expression of p53, increasing the expression level of Bax. Then caspase 3 was activated and cleaved PARP, inducing apoptosis (Fig. 3). At the same time, the expression of Bcl-2 was decreased. Our results confirmed that inhibiting polyamine synthesis in ESCC cells resulted in cell death due to apoptosis.

ODC activity displays two peaks during the entire cell cycle process, one associated with the G1/S transition and the other associated with the S/G2 and G2/M phases,^39^ suggesting that polyamines also play key roles in cell cycle progression. A number of studies reported that polyamine depletion arrests cells at the G1 phase.^40–42^ Other effects, including arrest at the S or G2/M phase, have been reported in different cell lines,^43–45^ suggesting that polyamine depletion affects the cell cycle in a cell-type specific manner. Our data showed that knocking down ODC expression arrested ESCC cells at the G2/M phase. Correspondingly, the percentage of cells in the S phase was decreased, with no significant difference at the G1 phase (Fig. 3b). Cell cycle progression is mediated by the family of cyclin-dependent kinases, which comprise a catalytic subunit and requisite positive regulatory subunits known as cyclins. Generally, positive regulation of CDK activity is mediated by the accumulation of cyclins, whereas negative regulation is achieved by phosphorylation of the catalytic subunit or by binding with CDK inhibitors, including p21 and p27.^46^ Cyclin B1 contributes to the transition of cells from the G2 to M phase and is inactivated by phosphorylation of CDK1.^47^ In ESCC, cyclin B1 was reported to be an independent prognostic factor in patients,^48^ indicating that cyclin B1 plays a key role in ESCC cell proliferation. Our results showed that polyamine depletion increased the expression of wildtype p53, which in turn directly down-regulated the transcription of *cyclin B1* mRNA and inactivated the CDK1/cyclin B1 complex by phosphorylation of CDK1. Moreover, as a transcription target of p53, p21 also can effectively inhibit the kinase activity of CDK1.^49^ Altogether, our data suggested that polyamine depletion arrested ESCC cells in the G2/M phase by decreasing the activity of the CDK1/cyclin B1 complex.

Based on our in vitro studies, we examined the role of ODC in ESCC progression in a xenograft mouse model. Our results showed that when ODC expression was blocked, ESCC cell proliferation was suppressed and apoptosis induced, causing reduced tumorigenesis in nude mice (Fig. 4). The data demonstrated that ODC plays a key role in ESCC progression, and could be a potential therapeutic target against ESCC.

DFMO, an FDA-approved drug for treatment of Trypanosomiasis, is an enzyme-activated irreversible inhibitor of ODC and acts to deplete polyamine pools. The antitumor effects of DFMO on various solid tumors, including colon cancer,^50^ skin cancer,^14^ pancreatic cancer,^51^ and breast cancer, has been reported in both preclinical and clinical studies.^52^ However, DFMO is generally cytostatic in mammalian cells, causing a reduction in the rate of proliferation in the absence of cell death.^53^ Based on our studies using *shODC*, DFMO’s effect on ESCC was examined. As expected, DFMO treatment not only suppressed proliferation, but also induced apoptosis of ESCC cells by inhibiting ODC activity (Fig. 5).

Despite results of research demonstrating impressive antitumor effects in preclinical studies, new therapies often fail to show significant efficacy in clinical trials, indicating that current methods, including immortalized cancer cell lines implanted into xenograft models, are suboptimal in predicting therapeutic efficacy. Major deficiencies of immortalized cancer cell line models are a lack of heterogeneity reflective of the original malignancy and an improper microenvironment.^54^ The PDX model is considered to be highly relevant to actual human tumor growth because the xenograft maintains the original molecular characteristics and heterogeneity.^55^ Thus, PDX models could have more predictive power for translating preclinical efficacy into clinical outcomes compared to xenograft models generated from established cell lines or genetically engineered mouse models. To obtain more preclinical data, we established 3 ESCC PDX model cell lines to test the effect of DFMO on ESCC growth. Results showed that DFMO treatment significantly inhibited the progression of ESCC without reducing mouse body weight. Moreover, IHC results reconfirmed that DFMO not only suppressed proliferation, but also induced apoptosis in ESCC cells, leading to the inhibition of ESCC progression. Notably, low doses of DFMO (2%) significantly inhibited the progression of ESCC PDX tumor growth whereas the high dose (4%) did not increase the effect, suggesting that in future clinical trials, increasing the DFMO dose will not enhance its antitumor effects.

In the present study, we reported high ODC expression in ESCC tissues compared with esophagitis or NAT. Our study also demonstrated that polyamine depletion not only suppressed proliferation, but also induced apoptosis of ESCC cells. Overall, the results of this study suggest that ODC is a promising target in ESCC therapy and that DFMO warrants further study in clinical trials to test its effectiveness against ESCC.

## Materials and methods

### Chemicals and reagents

A human ESCC tissue microarray (Catalog No. ES244) was purchased from US Biomax, Inc. (Rockville, MD) and DFMO (Catalog No. K100) was from AK Scientific, Inc. (Union City, CA). The primary antibodies against ODC (Catalog No. sc-390366), Bcl-2 (Catalog No. sc-7382), Bax (Catalog No. sc-7480), p21 (Catalog No. sc-6246), p27 (Catalog No. sc-1641), cyclin B1 (Catalog No. sc-245) and β-actin (Catalog No. sc-47778) were obtained from Santa Cruz Biotechnology (Santa Cruz, CA). The primary antibodies to detect cleaved PARP (Catalog No. 5625), phosphorylated ERKs (T202/Y204; Catalog No. 14474), E-cadherin (Catalog No. 3195), cleaved caspase 3 (Catalog No. 9661), p53 (Catalog No. 2524), phosphorylated CDK1 (Tyr15; Catalog No. 4539) were purchased from Cell Signaling Biotechnology (Beverly, MA) and the Ki-67 (Catalog No. MA5-14520) antibody was from Thermo Scientific (Fremont, CA). The *shODC* plasmids cloned into the lentiviral expression vector *pLKO.1* were obtained from the University of Minnesota (Minneapolis, MN). Human *shODC* full hairpin sequence is #1. 5′-CCGGGCCATATGGAAGACTAGGATACTCGAGTATCCTAGTCTTCCATATGGCTTTTTG-3′; #2. 5′-CCGGGCCGACGATCTACTATGTGATCTCGAGATCACATAGTAGATCGTCGGCTTTTTG-3′; #3. 5′-CCGGCCTTGTAAACAAGTATCTCAACTCGAGTTGAGATACTTGTTTACAAGGTTTTTG-3′; #4. 5′-CCGGGCGTCTATGGATCATTTAATTCTCGAGAATTAAATGATCCATAGACGCTTTTTG-3′; #5. 5′-CCGGCCTCCAGAGAGGATTATCTATCTCGAGATAGATAATCCTCTCTGGAGGTTTTTG-3′.

### Cell culture

Human ESCC cell lines (KYSE450, KYSE510) and the human embryonic kidney cell line (HEK293T) were purchased from American Type Culture Collection (ATCC; Manassas, VA). Each vial of frozen cells was thawed and maintained in culture for a maximum of 8 weeks. All cells were cytogenetically tested and authenticated before freezing. All cell culture conditions were performed following ATCC’s instructions.

### Lentiviral infection

The lentiviral packaging vectors (*pMD2.0G* and *psPAX*) were purchased from Addgene Inc. (Cambridge, MA). To prepare *shODC* lentiviral particles, the lentiviral vector and packaging vectors were transfected into HEK293T cells using iMFectin Poly DNA Transfection Reagent (GenDEPOT, Barker, TX) following the manufacturer’s suggested protocols. The transfection medium was changed at 8 h after transfection and then cells were cultured for 36 h. The lentiviral particles were harvested by filtration using a 0.45 µm sodium acetate syringe filter and then combined with 8 μg/ml of polybrane (Millipore, Billerica, MA) and infected overnight into 60% confluent KYSE450 and KYSE510 cells. The cell culture medium was replaced with fresh complete growth medium and after 24 h, cells were selected with 2 μg/ml of puromycine for an additional 24 h. The selected cells were used for experiments.

### ODC enzyme activity assay

ODC activity was measured as the release of CO_2_ from L-[1-C^14^] ornithine as previously described.^56^ Briefly, cell lysates were incubated at 37 °C for 60 min after addition of 0.5 µCi L-[1-C^14^] ornithine hydrochloride (Perkin Elmer, Waltham, MA), 40 mM sodium phosphate buffer (pH 7.8) containing 0.64 mM pyridoxal phosphate, 0.8 µM EDTA and 8 mM dithiothreitol. The reaction was terminated by addition of 10% trichloroacetic acid. The released ^14-^CO_2_ was soaked in scintillation solution (Research Products International Corp., Mount Prospect, IL) and radioactivity was measured by scintillation counter (LS6500, Beckman Coulter, Fullerton, CA) and the results are expressed as percentage of the control, which was set at 100%.

### Cell proliferation assay

Cells (1 × 10^3^ per well) were seeded into 96-well plates. After overnight incubation, cells were treated with different concentrations of DFMO (0, 0.25, 0.5, 1, and 1.5 mM), and incubated for 24, 48, or 72 h. Then 20 μl Cell Titer 96 Aqueous One Solution (Promega Corporation, Madison, WI) were added and cells incubated for another 1 h. Absorbance was read at 492 nm.

### Anchorage-independent cell growth assay

Cells (8 × 10^3^ per well) suspended in complete growth medium (Eagle’s basal medium supplemented with 10% fetal bovine serum (FBS) and 1% antibiotics) were added to 0.3% agar with different doses of DFMO in a top layer over a base layer of 0.6% agar with the same doses of DFMO. The cultures were maintained at 37 °C in a 5% CO_2_ incubator for 3 weeks and then colonies were counted under a microscope using the Image-Pro Plus software (v.6.0) program (Media Cybernetics, Inc. Rockville, MD).

### Analysis of apoptosis and cell cycle by flow cytometry

For analysis of apoptosis, cells (2 × 10^5^ cells per well) were seeded into 6-well plates and cultured for 24 h, and then exposed to DFMO (0, 0.25, 0.5, 1, or 1.5 mM) for 72 h. Cells were trypsinized and washed twice with cold phosphate-buffered saline (PBS) solution, and then resuspended with PBS and incubated for 5 min at room temperature with annexin V-fluorescein isothiocyanate (FITC) plus propidium iodide. Cells were analyzed using a FACS Calibur flow cytometer (BD Biosciences, San Jose, CA). For cell cycle analysis, cells were treated with DFMO, harvested and washed twice with PBS, and fixed with cold 70% ethanol overnight at −20 °C. Stained cells were detected and quantified by flow cytometry.

### Western blot analysis

Protein concentrations of *ShODC* or DFMO-treated ESCC cell lysates were determined using a protein assay kit (Bio-Rad Laboratories, Inc. Hercules, CA). Total proteins (20 to 100 μg) were separated by SDS-PAGE and transferred onto a polyvinylidene difluoride membrane (Millipore, Billerica, MA). After blocking in 5% non-fat milk, the membranes were probed with specific primary antibodies overnight at 4 °C, then washed 3 times with TBS-Tween 20 followed by incubation at room temperature 1 h with a horseradish peroxidase (HRP)-conjugated secondary antibody. The protein bands were visualized with a chemiluminescence reagent (GE Healthcare Biosciences, Pittsburgh, PA).

### In vivo studies using a xenograft mouse model

NU/NU nude female mice (6 weeks old; Charles River, Chicago, IL) were randomly divided into three groups of four each and inoculated in the right flank with KYSE450-*shMock*, KYSE450-*shODC #2* or KYSE450-*shODC #4* lentiviral-infected cells (3 × 10^6^ cells permouse). Mice were maintained under “specific pathogen-free” conditions based on the guidelines established by the University of Minnesota Institutional Animal Care and Use Committee. Tumor volume and body weight were measured once a week. Tumor volume was calculated from measurements of three diameters of the individual tumor base using the following formula: tumor volume (mm^3^) = (length × width × height × 0.52). Mice were monitored until tumors reached 1000 mm^3^ total volume, at which time mice were euthanized and tumors extracted.

### Effect of DFMO on a human ESCC PDX mouse model

Human ESCC tumor fragments were obtained from three male ESCC patients who underwent esophagectomy at the First Affiliated Hospital of Zhengzhou University (Zhengzhou, Henan, China) without any neo-adjuvant therapy (Supplementary Table 1). In China, ESCC is much more common in males than in females. According to the epidemiological investigation of ESCC incidence in 2003, ESCC incidence in males is 19.68/100,000 compared to 9.85/100,000 in females. Thus for these initial studies, we used tumor fragments from male patients only, but will examine tumors from female patients in future studies. A frozen section was stained with H&E and evaluated to confirm the diagnosis. A fresh ESCC tissue fragment was collected and transferred at 4 °C in FBS-free RPMI-1640 medium with antibiotics. Within 2 h of surgical resection, the tumor tissue was trimmed, cut into 3-5 mm pieces and implanted subcutaneously in anesthetized 6–8 week old female C.B-17 severe combined immunodeficient mice (Vital River Laboratories Co., Ltd., Beijing, China). Once mass formation reached about 1500 mm^3^, mice of this first generation of xenografts (named G1) were sacrificed and the tumors were passaged and expanded for two more generations (named G2 and G3). When G3 tumors reached an average volume of 250 mm^3^, mice were divided into three groups (seven mice per group) randomly and treated with vehicle or 2% (v/v) or 4% (v/v) DFMO in drinking water, respectively. The concentration of DFMO (2% v/v in drinking water) was founded on a previous report.^13^ Based on average mouse daily water intake (4 ml per day), 80 mg (4 g/kg body weight) DFMO would be consumed by a mouse per day. At the same time, we also set a higher concentration group (4%) for comparison. Tumor volume and body weight were measured every three days. Tumor volume was calculated from measurements of three diameters of the individual tumors using the following formula: tumor volume (mm^3^) = (length × width × height × 0.52). Mice were monitored until tumors reached 1000 mm^3^ total volume, at which time mice were euthanized and tumors extracted. Seven animals per group were recruited to achieve statistical significance. Mice were randomly grouped and treated without investigator blinding.

The patients who donated the primary tumors were completely informed and provided written consent. Human tissue collection and use protocols were approved by the ethics committee of Zhengzhou University (Zhengzhou, Henan, China. Access No.: CUHCI2016012).

### IHC staining

Tissue sections were deparaffinized in xylene and microwaved in 10 mM citrate buffer (pH 6.0) to unmask the epitopes. Endogenous peroxidase activity was blocked by incubation for 10 min with 0.03% hydrogen peroxide in methanol. Immunohistochemical staining was performed using the indirect avidin biotin-enhanced HRP method according to the manufacturer’s instructions (Vector Laboratories, Burlingame, CA). After developing with 3,3′-diaminobenzidine, all sections were counterstained with hematoxylin and observed by microscope (200×). Quantitative analysis of IHC staining was performed using the Image-Pro Plus software (v.6.0) program (Media Cybernetics, Inc. Rockville, MD).

### Statistical analysis

All quantitative results are expressed as mean values ± S.D. of triplicate values from three independent experiments. Significant differences were compared using the Student’s *t* test (single tailed). A *p* value of <0.05 was considered to be statistically significant.

## Electronic supplementary material

Supplemental Figure Legend

Supplemental Figure 1

Supplemental Figure 2

Supplemental Figure 3

Supplemental Table 1

Supplemental Table 2

## Electronic supplementary material

Supplementary Information accompanies the paper on the *npj Precision Oncology* website (doi:10.1038/s41698-017-0014-1).
