# Supplementary material for: Targeting ornithine decarboxylase (ODC) inhibits esophageal squamous cell carcinoma progression
Source: NPJ Precis Oncol. 2017 Apr 27;1:13. doi: 10.1038/s41698-017-0014-1 (PMC5859467; doi:10.1038/s41698-017-0014-1)
Supplement: Supplementary file 1 — Supplemental Figure Legend [file 41698_2017_14_MOESM1_ESM.docx]

**Supplementary Figure Legends**

**Figure S1. ODC IHC staining of an ESCC tissue array.** The array contains a total of 144 evaluable cases, including 110 ESCC, 15 esophagitis and 19 adjacent normal tissues.

**Figure S2. *shODC* suppresses tumor-forming ability of ESCC cells.** At 56 days after inoculation, when the tumor volume of the *shMock* group reached 1,000 mm^3^, all experimental mice were sacrificed. Compared with the *shMock* group, the *shODC-*transfected KYSE450 cells formed almost no xenograft tumors in nude mice.

**Figure S3. DFMO inhibits ESCC progression in the EG5 and EG7 PDX models**

Compared with vehicle, DFMO treatment significantly decreased the average weight of EG5 (a) and EG37 (b) PDX tumors. All data are shown as mean values ± S.D. and the asterisks (*, **) indicate a significant (*p* < 0.05, *p* < 0.01, respectively) difference between the DFMO-treated and vehicle control groups.
