# Supplementary figures and images for: Targeting ornithine decarboxylase (ODC) inhibits esophageal squamous cell carcinoma progression
Source: NPJ Precis Oncol. 2017 Apr 27;1:13. doi: 10.1038/s41698-017-0014-1 (PMC5859467; doi:10.1038/s41698-017-0014-1)

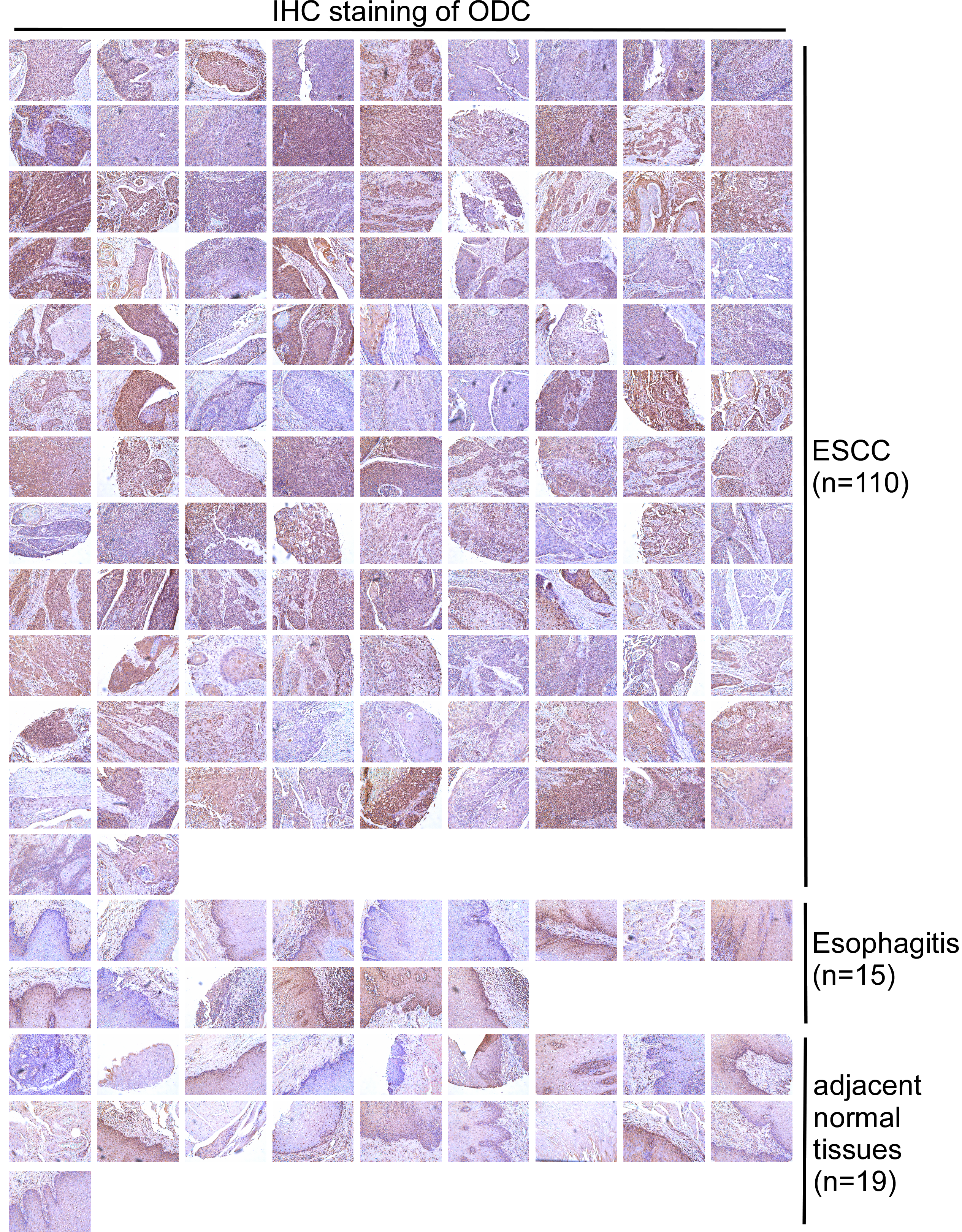

Supplement: Supplementary file 2 — Supplemental Figure 1 [file 41698_2017_14_MOESM2_ESM.tif]

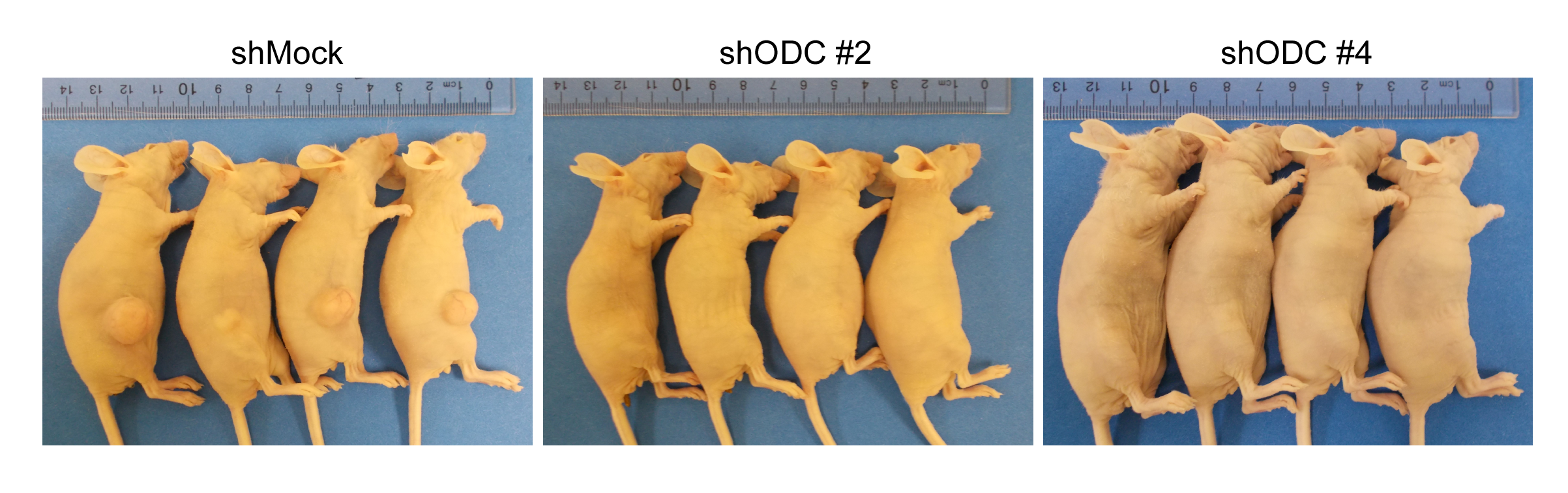

Supplement: Supplementary file 3 — Supplemental Figure 2 [file 41698_2017_14_MOESM3_ESM.tif]

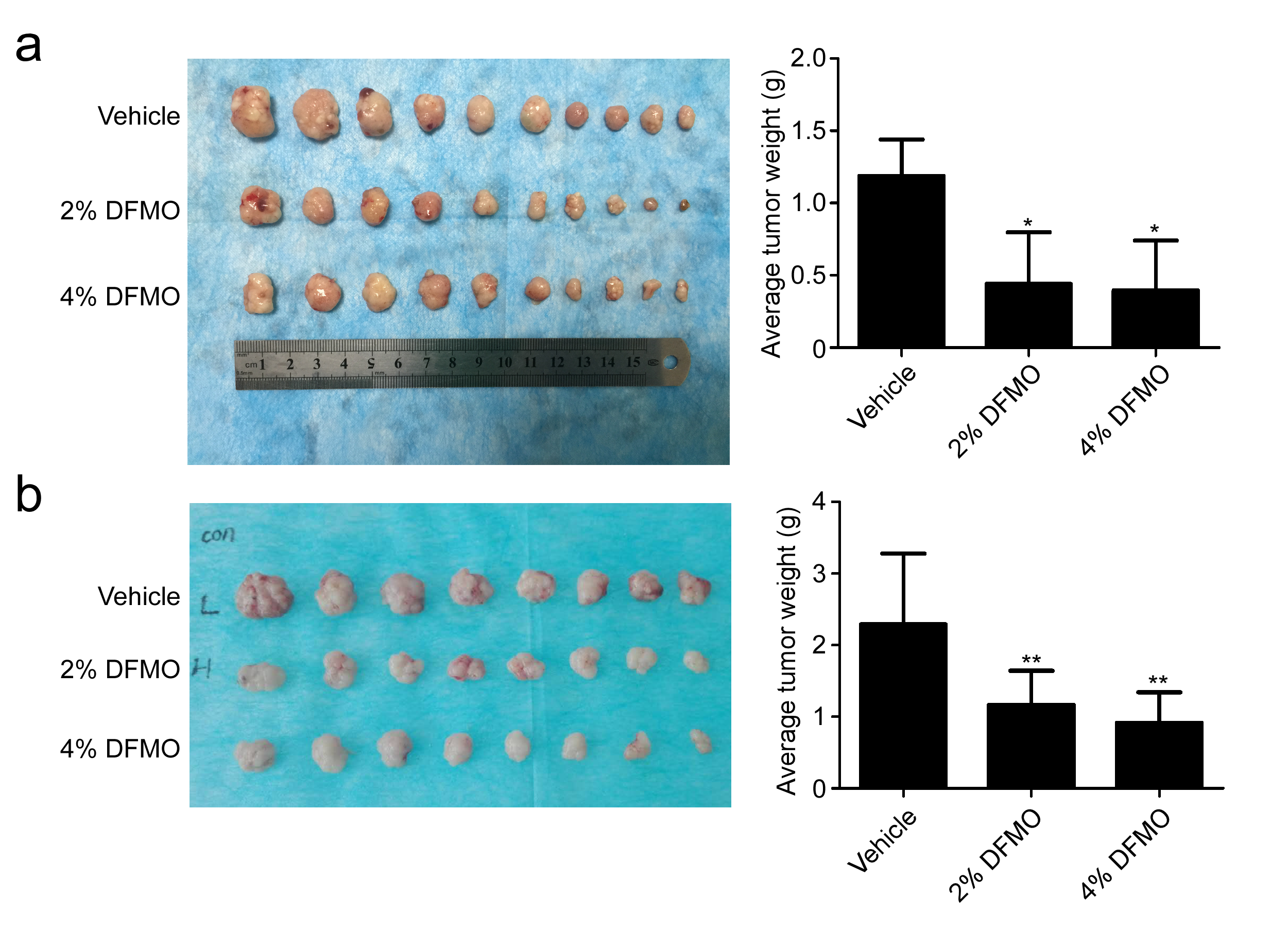

Supplement: Supplementary file 4 — Supplemental Figure 3 [file 41698_2017_14_MOESM4_ESM.tif]
