# Supplementary material for: Targeting ornithine decarboxylase (ODC) inhibits esophageal squamous cell carcinoma progression
Source: NPJ Precis Oncol. 2017 Apr 27;1:13. doi: 10.1038/s41698-017-0014-1 (PMC5859467; doi:10.1038/s41698-017-0014-1)
Supplement: Supplementary file 5 — Supplemental Table 1 [file 41698_2017_14_MOESM5_ESM.docx]

**Supplementary Table 1.** Clinical characteristics of the original ESCC tissues used in the PDX model.

| **Model ID** | **Gender** | **Age (years)** | **Source** | **Histology** | **Histological grade** | **TNM stage** |
| --- | --- | --- | --- | --- | --- | --- |
| EG20 | male | 46 | Primary | ESCC | II | T_2_N_0_M_0_ |
| EG5 | male | 61 | Primary | ESCC | II | T_2_N_0_M_0_ |
| EG37 | male | 69 | Primary | ESCC | III | T_3_N_0_M_0_ |
