# Supplementary material for: Targeting ornithine decarboxylase (ODC) inhibits esophageal squamous cell carcinoma progression
Source: NPJ Precis Oncol. 2017 Apr 27;1:13. doi: 10.1038/s41698-017-0014-1 (PMC5859467; doi:10.1038/s41698-017-0014-1)
Supplement: Supplementary file 6 — Supplemental Table 2 [file 41698_2017_14_MOESM6_ESM.docx]

**Supplementary Table 2.** Expression of ODC in ESCC tissues

| **Parameter** | | **n** | **ODC expression (IOD sum)** | ***p* value** |
| --- | --- | --- | --- | --- |
| Gender | | | | |
|  | Male | 76 | 2348 ± 139 | 0.77 |
|  | Female | 34 | 2049 ± 205 |  |
| Age (years) | | | | |
|  | ≤ 60 | 69 | 2294 ± 151 | 0.67 |
|  | > 60 | 41 | 2191 ± 179 |  |
| Histological grade | | | | |
|  | I | 37 | 2250 ± 210 | 0.25 |
|  | II | 40 | 2040 ± 184 |  |
|  | III | 33 | 2514 ± 203 |  |
| Lymph node metastasis | | | | |
|  | Negative | 64 | 2042 ± 136 | 0.01* |
|  | Positive | 46 | 2547 ± 194 |  |
|  | Negative | 64 | 2042 ± 136 |  |
| TNM stage | | | | |
|  | II | 73 | 2007 ± 125 | 0.02* |
|  | III | 37 | 2597 ± 219 |  |
| *Indicates statistical significance (*p* < 0.05) | | | |  |
